# Incarcerated Left Paraduodenal Hernia as a Rare Cause of Acute Abdomen: A Case Report and Literature Review

**DOI:** 10.7759/cureus.75878

**Published:** 2024-12-17

**Authors:** Shirish Bhagvat, Najmeh Mirkhushal, Shalmali Dharmadhikari, Abhipray P Ramteke, Rajalakshmi Venkateswaran

**Affiliations:** 1 General Surgery, Grant Medical College and Sir JJ Group of Hospitals, Mumbai, IND

**Keywords:** contrast enhanced computed tomography, emergency diagnostic laparoscopy, incarcerated pdh, internal hernia, intestinal obstruction, para- duodenal hernias

## Abstract

Internal hernias are one of the rare causes of intestinal obstruction and usually is the diagnosis of exclusion. Para-duodenal hernias (PDH) are rare congenital disorders that occur due to malrotation of the midgut in the embryonic phase of development. They can be asymptomatic or can present as an incarcerated, strangulated, or even obstructed internal hernia. It is difficult to diagnose clinically and often requires supportive radiological investigations. Herewith, we present a case of a 30-year-old male who presented with a short history of persistent pain in the abdomen. Contrast-enhanced computed tomography (CECT) showed the presence of an uncomplicated left para-duodenal hernia. Emergency diagnostic laparoscopy was done in view of abdominal tenderness, and a diagnosis of incarcerated PDH was made. The PDH was then tackled laparoscopically. We present this case to highlight the importance of suspecting a PDH with complications in an acute abdomen and the need to intervene surgically based on clinical judgment.

## Introduction

Internal hernias are a rare pathology that can be due to congenital or acquired factors, with the latter being the most common. They have been classified into eight subtypes by Welch based on the topography of the contents and the anatomical location of the orifice [1]. The eight subtypes are para-duodenal hernias (PDH), foramen of winslow hernia, pericaecal hernia, trans sigmoid mesocolon hernia, trans-mesenteric hernia, trans-omental hernia, pelvic hernia and supra-vesical hernia. Another classification system has been proposed, which classifies them into three variants depending on the type of orifice: natural, peritoneal fossae or recess, or an abnormal opening in the mesentery or peritoneal ligament [1].

The most common presentation of this condition is pain in the abdomen, mostly vague, dull, aching chronic discomfort, and, at times, acute worsening abdomen pain. The incidence of strangulation or obstruction in these cases has been seen only in 0.2% to 0.9% in the literature but is also significant as this is one of the most overlooked causes of an acute abdomen [2]. The index case is a patient who presented with an acute presentation of an internal hernia, which is mostly a diagnosis of exclusion; hence, it is significant to discuss. 

## Case presentation

**Figure 1:**
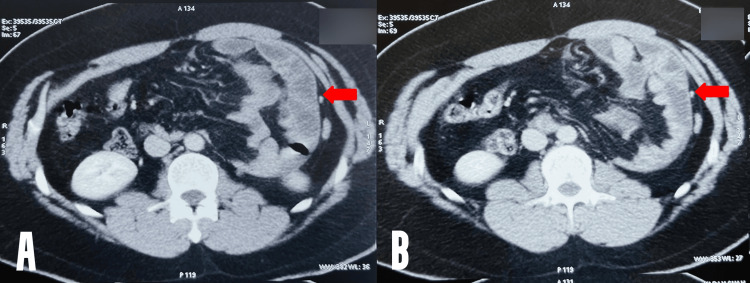
CECT images of the case. A, B: Red arrow indicates the sac with the dilated jejunal loops in it herniating into the left mesocolon. The jejunal loops show maintained vascularity.

A 30-year-old male patient presented to the outpatient clinic with severe, progressive, dull, aching, generalized abdomen pain for two days. There was no associated abdominal distension, vomiting, or obstipation. On examination, the patient had a pulse rate of 116 beats per minute and blood pressure of 130/86mmHg. An abdomen examination revealed tenderness in the umbilical region, left hypochondrium, and left lumbar region, with no other significant findings such as distension, guarding, or rigidity. Per rectal examination, it was unremarkable. X-ray of the abdomen revealed the presence of a few dilated bowel loops of bowel in the left lumbar region. There was no free gas under both domes of the diaphragm to suggest a hollow viscus perforation. A contrast-enhanced computed tomography (CECT) was performed, which revealed the presence of a left PDH (Figure 1).

A CT angiography was also done, which ruled out a vascular compromise/ bowel ischemia. The patient was initially managed conservatively for the first 36 hours. However, he continued to have the same pain severity with unstable vitals. Hence, he was planned for a diagnostic laparoscopy. 

**Figure 2:**
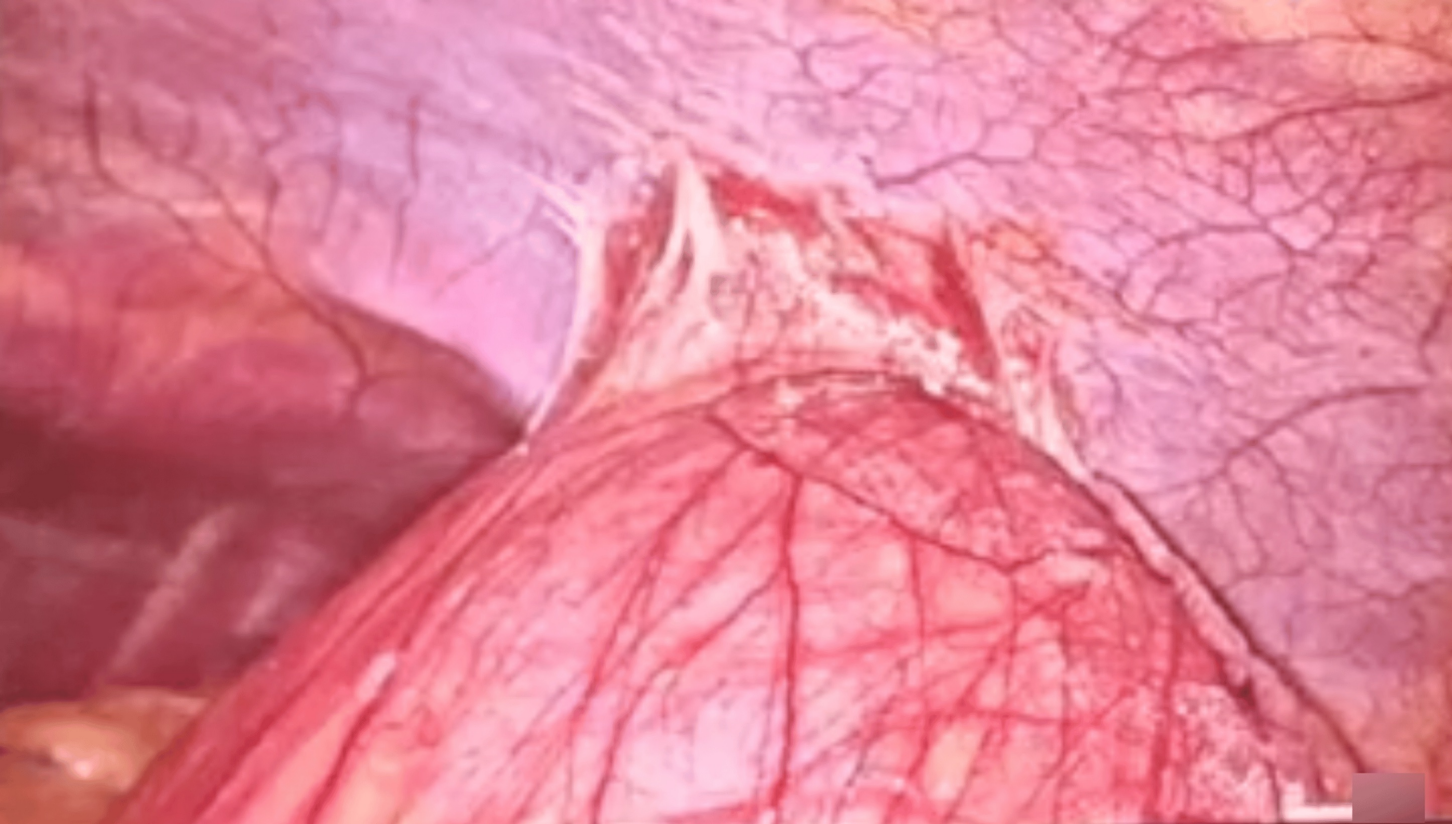
Figure showing the sac adhered to the parietal peritoneum.

The surgery was performed under general anesthesia. The surgeon operated from the right side of the patient. A 10 mm camera port was inserted infra-umbilically. Two 5mm working ports were inserted 5 cm below the costal margins on either side of the midline. An assistant port was inserted along the left midclavicular line, along the line of the umbilicus, for retraction. After a diagnostic laparoscopy, the sac was seen to adhere to the parietal wall (Figure 2). 

**Figure 3:**
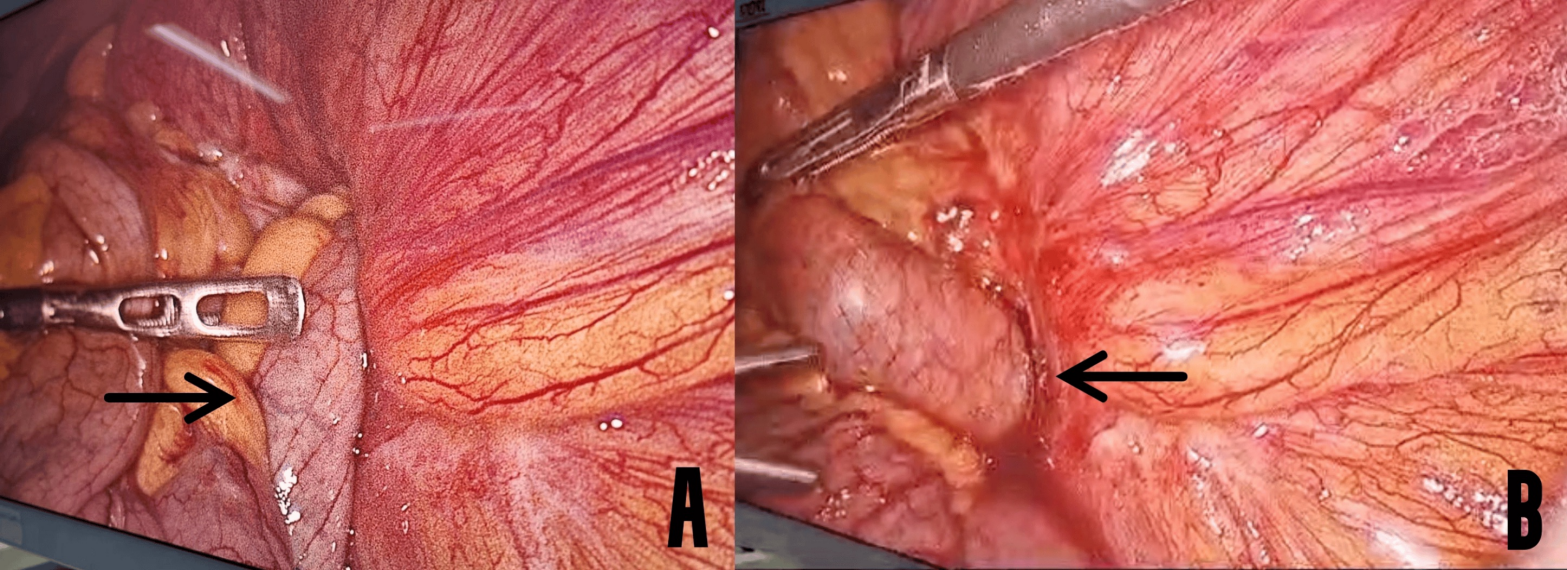
Figure showing herniation of the bowel loops into the transverse mesocolon. A: Jejunal loops entering into the hernia defect with a black arrow indicating the jejunal loops. B: Figure showing the defect in the mesocolon, which is indicated by the black arrow.

The bowel loops were then traced to identify the segment that was entrapped in the hernia sac. The proximal jejunal loops were seen entering through a defect in the transverse mesocolon, going towards the left hypochondrium (Figure 3A). The contents were carefully manipulated through the defect using a blunt instrument (Figure 3B). 

**Figure 4:**
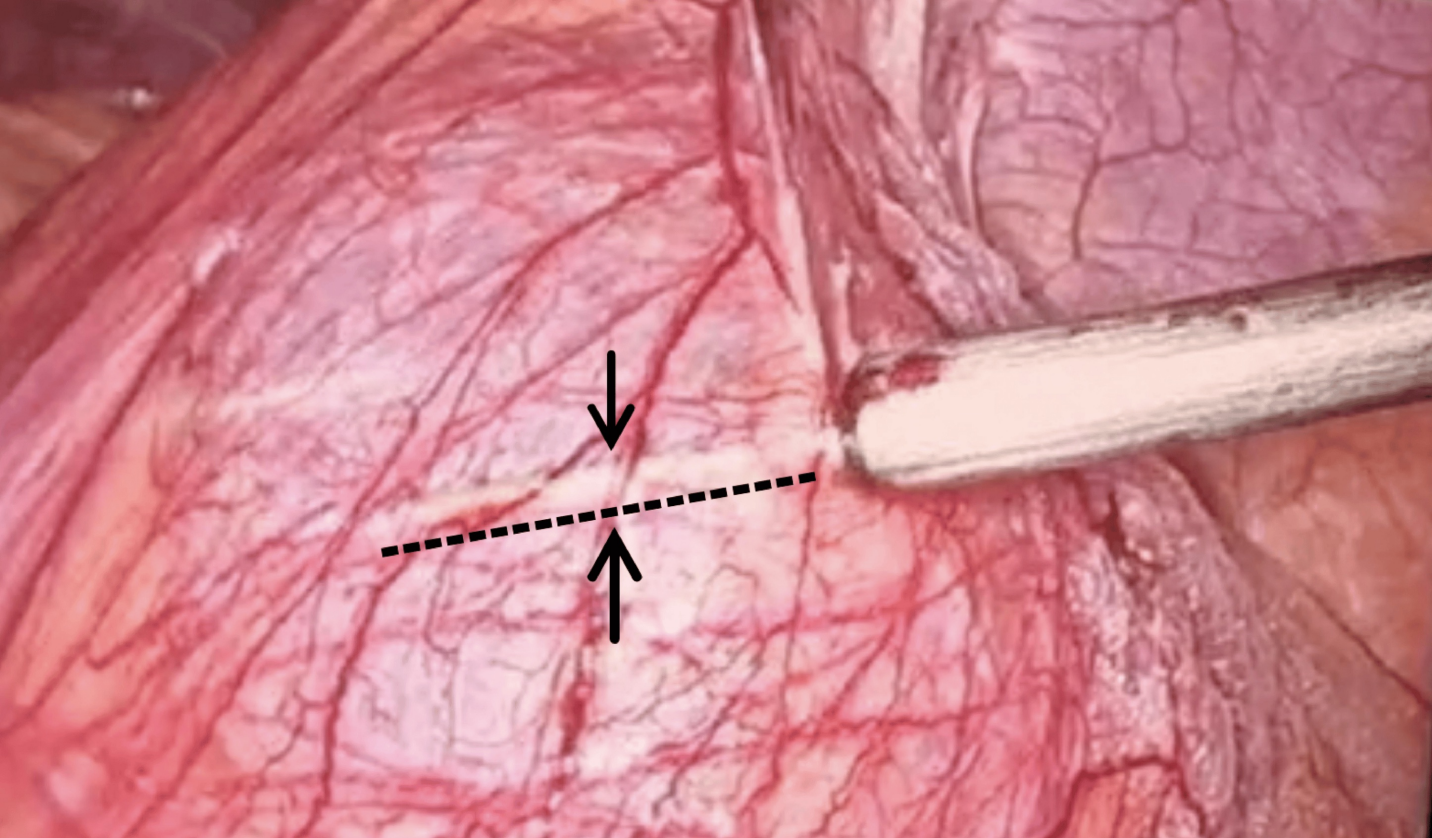
Figure showing the needle of the aspirator along which the sac was opened later. The trajectory of the needle can be visualized through the wall of the sac just above and parallel to the black dotted line and in between the two black arrows.

During delivery of the jejunum through the defect, it was observed that a part of the jejunum was densely adhered to the sac, and it was not feasible to deliver through normal traction, thus suggesting an incarcerated let PDH. Hence, a decision was taken to open the sac and perform adhesiolysis. The fundus of the sac was palpated, and the part free of the bowel was identified. The sac was punctured at that site (of maximum transparency), and clear fluid was aspirated. The sac was incised along the line of the puncture needle, which was visible through the sac wall to ensure no bowel was incised while opening the sac (Figure 4). The sac was incised using monopolar diathermy.

The sac was opened, and adhesions between the bowel wall and the sac were taken down using a bipolar device. The jejunum was safely delivered through the defect. The excess sac was excised, and the defect in the mesocolon was closed with a Polydioxanone suture. The surgery was uneventful. On the postoperative day 1, the patient was started on a diet, which he tolerated well. He was subsequently discharged the next day. Serial follow-up on an outpatient basis revealed complete relief from pain.

## Discussion

Internal hernias account for less than 1% of cases of intestinal obstruction in adults, and PDH makes up nearly 50-55% of all internal hernias. Left-sided PDHs are more common than right-sided ones, with an incidence of around 75%, and are usually congenital [3]. Internal hernias most frequently follow acquired factors such as trauma, ischemia, inflammation, and abdominal surgeries such as liver transplants and bariatric surgeries (Roux en Y gastric bypass) [4,5]. They are usually clinically silent or present with vague symptoms such as chronic upper abdominal pain, reflux, and gastritis. Hence, these patients are evaluated and managed as irritable bowel syndrome, gastritis, reflux disease, or biliary gastritis [6]. Obstruction or incarceration of these hernias is usually the final stage of the condition. 

In the left PHD, herniation occurs through the Landzert fossa, which is present to the left of the fourth part of the duodenum and extends posteriorly to the descending colon [7]. The small bowel travels posterior to the Ligament of Treitz [8], herniates through the Lanzert fossa, and lies posterior to the left portion of the transverse mesocolon. The anterior wall of the sac and the neck contains the inferior mesenteric vein and branches of the left colic artery [7]. Hence, in cases where the sac needs to be opened to release its contents, the sac should never be opened at the neck, which was followed in the index case. In the present case, since it was done laparoscopically and the site of adhesion could not be visualized from within the sac, the decision to pass a needle aspirator through the most transparent part of the sac at its fundus was taken. After that, the sac was opened along the line where the needle could be visualized through the sac. 

X-rays in such cases usually show an abnormal clustered loop of bowel in the left upper quadrant of the abdomen. Contrast-enhanced CT scans are best to diagnose these hernias wherein the hernial contents are seen looped into a sac at the level of the Ligament of Treitz and behind the pancreas [8,9]. They can help to diagnose an obstructed internal hernia with or without an ischaemic bowel but can miss out on strangulated or incarcerated ones rarely, as seen in the present case [8]. Coeliac artery angiography can show a displaced spleen, and superior mesenteric artery angiography can show jejunal arteries displaced to the left and upward [9].

The abdominal pain in such cases can be intermittent as well because the contents freely move in and out of the sac. Significant pain develops when the contents get adhered or entrapped in the sac, as happens in any hernia case. Incarceration of left PDH occurs in 50% of untreated cases [10]. Hence, asymptomatic ones should also be operated on electively to prevent strangulation or bowel ischemia. In a patient with PDH who previously had intermittent symptoms but now presents with persistent pain, the possibility of an incarcerated or strangulated PDH should always be considered. The CT scan in the index case also could not pick up an incarcerated PDH, but the patient was taken up for emergency diagnostic laparoscopy based on clinical suspicion, which eventually proved to be right. 

Minimal invasive surgery has shown to be advantageous in these cases in terms of reduced post operative pain and early recovery after surgery as highlighted by Shadhu K et all in their review on 5 cases [11]. In this case, the key principles followed while operating laparoscopically were meticulous adhesiolysis, reduction of contents into the abdomen, sac excision, and closure of the mesocolon defect. 

## Conclusions

Para-duodenal hernias, no doubt, are rare causes of intestinal obstruction but need to be tackled surgically as more than half of them end up with intestinal obstruction in later stages. Any patient with an acute abdomen, where CT scans confirm the diagnosis, should be suspected to have an incarcerated or an obstructed internal hernia unless there is an alternative diagnosis. Due to their wide spectrum of presentation, any diagnosed case of PDH who develops worsening abdominal pain should be suspected to have incarceration or strangulation and should be surgically explored at the earliest. Minimal-invasive surgery is beneficial in most cases, but it should be borne in mind that the sac should never be opened at the neck as it can result in injury to named vessels.
